# Functional and *in silico* analysis of ATP8A2 and other P4-ATPase variants associated with human genetic diseases

**DOI:** 10.1242/dmm.050546

**Published:** 2024-04-24

**Authors:** Eli Matsell, Jens Peter Andersen, Robert S. Molday

**Affiliations:** ^1^ Department of Biochemistry & Molecular Biology, University of British Columbia, Vancouver, British Columbia V6T 1Z3, Canada; ^2^ Department of Biomedicine, Aarhus University, 8000 Aarhus, Denmark

**Keywords:** *In silico* protein stability, Missense mutations, Neurodevelopmental disease, P4-ATPases, Protein misfolding, Disease mechanisms

## Abstract

P4-ATPases flip lipids from the exoplasmic to cytoplasmic leaflet of cell membranes, a property crucial for many biological processes. Mutations in P4-ATPases are associated with severe inherited and complex human disorders. We determined the expression, localization and ATPase activity of four variants of ATP8A2, the P4-ATPase associated with the neurodevelopmental disorder known as cerebellar ataxia, impaired intellectual development and disequilibrium syndrome 4 (CAMRQ4). Two variants, G447R and A772P, harboring mutations in catalytic domains, expressed at low levels and mislocalized in cells. In contrast, the E459Q variant in a flexible loop displayed wild-type expression levels, Golgi–endosome localization and ATPase activity. The R1147W variant expressed at 50% of wild-type levels but showed normal localization and activity. These results indicate that the G447R and A772P mutations cause CAMRQ4 through protein misfolding. The E459Q mutation is unlikely to be causative, whereas the R1147W may display a milder disease phenotype. Using various programs that predict protein stability, we show that there is a good correlation between the experimental expression of the variants and *in silico* stability assessments, suggesting that such analysis is useful in identifying protein misfolding disease-associated variants.

## Teaser

**Summary:** Biochemical studies and *in silico* protein stability analyses reveal molecular mechanisms underlying the neurodevelopmental disorder CAMRQ4 associated with missense mutations in ATP8A2 and genetic diseases linked to other P4-ATPase lipid flippases.

**Research Simplified**

Cerebellar ataxia, impaired intellectual development and disequilibrium syndrome 4 (CAMRQ4) is a rare neurodevelopmental disorder that is inherited. Patients with CAMRQ4 often suffer from lack of muscle coordination and intellectual disability. The cause of CAMRQ4 is linked to some variants of the gene that codes for the ATP8A2 protein – a protein that actively transports important molecules called phospholipids across cell membranes to maintain proper cellular functioning. Understanding how certain variants of ATP8A2 cause CAMRQ4 is important to develop therapeutic strategies.

The authors of this study investigated four disease-associated ATP8A2 variants in human cells. Two of these genetic variants resulted in significantly lower production of the protein, with a disrupted and unstable protein structure, compared to that of non-mutated ATP8A2. These protein variants also did not localise within the correct part of the cell for their proper function. Using software programmes, the authors could accurately predict twelve other known CAMRQ4-associated ATP8A2 variant proteins that are produced at low levels and are structurally unstable.

This study establishes complementary tools to identify ATP8A2 variants that potentially cause CAMRQ4. Investigating this further could help researchers develop therapeutics for CAMRQ4 that help improve ATP8A2 function.

## INTRODUCTION

**Fig. 1.**
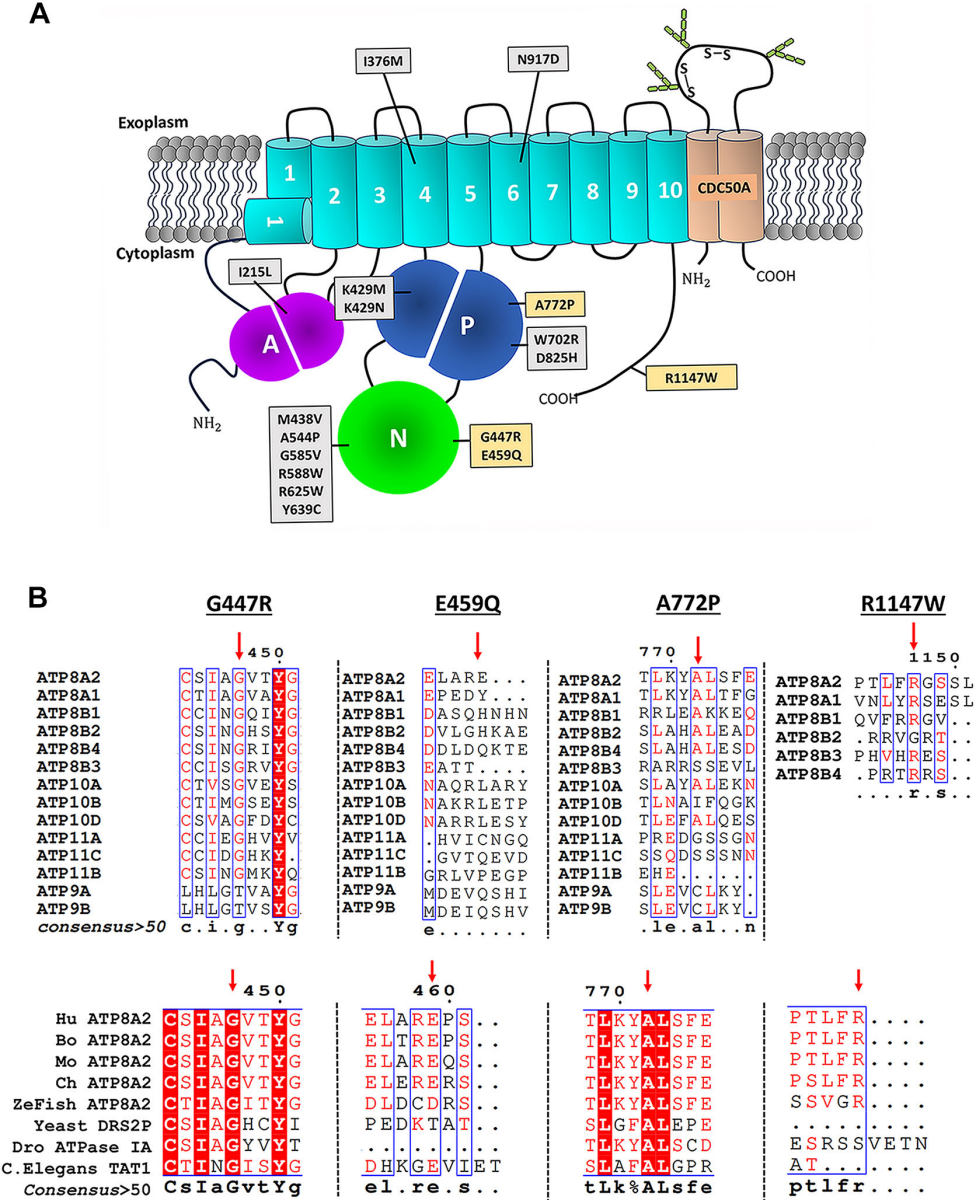
**ATP8A2 disease variants and sequence conservation.** (A) Topological model of ATP8A2 showing the location of various disease variants. The α-subunit ATP8A2 consists of a transmembrane domain (teal) with ten membrane spanning segments, an actuator domain (‘A’, purple), a phosphorylation domain (‘P’, blue) and the nucleotide-binding domain (‘N’, green). The missense variants G447R, E459Q, A772P and R1147W are highlighted and the subject of this study. (B) Sequence alignments of segments containing the G447, E459, A772 and R1147 residues in human P4-ATPases and ATP8A2 from various species (human, Q9NTI2; *Bos taurus*, C7EXK4; *Mus musculus*, P98200; *Gallus gallus*, A0A1D5P6U3; *Danio rerio*, A0A8M1RFW8; and the yeast homolog Drs2p, P39524). Alignments were conducted using the ESPript program (https://espript.ibcp.fr/ESPript/ESPript/), and the consensus residues are displayed below each alignment.

P4-ATPases constitute a subfamily of P-type ATPases that use the energy from ATP hydrolysis to flip phospholipids from the exoplasmic to cytoplasmic leaflet of cell membranes (Andersen et al., 2016; Folmer et al., 2009a; Lopez-Marques et al., 2014; Palmgren et al., 2019; Sebastian et al., 2012). This generates and maintains transmembrane lipid asymmetry, a property that is crucial for cellular processes such as vesicle transport, phagocytosis, apoptosis, sperm maturation, blood coagulation, synaptic modeling and cell migration, among others (Bevers and Williamson, 2016; Hankins et al., 2015; Lee et al., 2015; Li et al., 2021; Segawa and Nagata, 2015). P4-ATPases consist of a catalytic or α-subunit comprising a transmembrane domain that functions in the translocation of lipids across the membrane, a nucleotide or N-domain involved in nucleotide binding, a phosphorylation or P-domain with a conserved DKTGT/S motif that undergoes transient phosphorylation, and an actuator or A-domain with a conserved DGET motif that is responsible for dephosphorylation of the phosphorylated intermediate as part of the Post–Albers reaction cycle (Albers, 1967; Andersen et al., 2016; Dyla et al., 2020; Post et al., 1972) (Fig. 1). In addition, most P4-ATPases form a heterodimer with an accessory or β-subunit that is essential for the proper folding of the active complex and its exit from the endoplasmic reticulum (ER) (Bryde et al., 2010; Coleman and Molday, 2011; Saito et al., 2004; van der Velden et al., 2010b). P4-ATPases transport a variety of membrane lipids, including phosphatidylserine (PS), phosphatidylethanolamine, phosphatidylcholine and glycosphingolipids (Coleman et al., 2009; Roland et al., 2019; Shin and Takatsu, 2019).

The human genome encodes 14 distinct P4-ATPases (ATP8A1, ATP8A2, ATP8B1, ATP8B2, ATP8B3, ATP8B4, ATP9A, ATP9B, ATP10A, ATP10B, ATP10D, ATP11A, ATP11B and ATP11C) based on their α-subunit and two β-subunits referred to as CDC50A and CDC50B (also known as TMEM30A and TMEM30B). A gene encoding a third subunit CDC50C (TMEM30C) is present in the mouse genome, but it is considered as a pseudogene in humans. The α-subunits differ not only in their amino acid sequence, but also in their tissue expression, subcellular distribution, substrate specificity and regulatory properties. CDC50A is the predominant β-subunit as it interacts with 12 different P4-ATPases (van der Velden et al., 2010b; Wang et al., 2018). More recently, the high-resolution structures of three human P4-ATPases (ATP8A1, ATP8B1 and ATP11C) in association with CDC50A have been determined in various states by either X-ray crystallography or cryo-electron microscopy (Cheng et al., 2022; Dieudonne et al., 2022; Hiraizumi et al., 2019; Nakanishi et al., 2020a,b; Timcenko et al., 2021). These studies together with site-directed mutagenesis have provided important insight into the protein conformational transitions that occur as part of the reaction cycle, lipid-binding sites, subunit interactions, lipid transport pathways and regulatory mechanisms (Lyons et al., 2020; Mogensen et al., 2023; Vestergaard et al., 2014).

The importance of P4-ATPases in human cell physiology is evident from the finding that defects in the genes encoding numerous phospholipid flippases are associated with severe hereditary diseases. Mutations in the gene encoding ATP8A2 have been linked to the neurodevelopmental disorder known as cerebellar ataxia, impaired intellectual development and disequilibrium syndrome 4 (CAMRQ4) (Heidari et al., 2021; Martin-Hernandez et al., 2016; McMillan et al., 2018; Onat et al., 2013). Genetic defects in *ATP8B1* cause progressive familial intrahepatic cholestasis 1 (PFIC1) or benign recurrent intrahepatic cholestasis 1 (BRIC1) (Bull et al., 1998; Folmer et al., 2009b). Mutations in *ATP11C* are responsible for congenital hemolytic anemia (Arashiki et al., 2016; van Dijk et al., 2023), and mutations in *ATP11A* have been linked to a hypomyelinating leukodystrophy (Segawa et al., 2021) and hearing loss (Pater et al., 2022). Many of these disease phenotypes have been replicated in knockout or transgenic mice (Coleman et al., 2014; Segawa et al., 2021; Siggs et al., 2011; Stapelbroek et al., 2009; Zhu et al., 2012). Various P4-ATPases and CDC50A have also been implicated in complex disorders including cancer, Alzheimer's disease, Parkinson's disease, atherosclerosis and diabetes, among others (Ennishi et al., 2020; Kengia et al., 2013; Martin et al., 2020).

Previously, we characterized several missense mutations in ATP8A2 and showed that a number of these mutations cause a loss in function through protein misfolding or the loss in a key residue required for the function of ATP8A2 as an ATP-dependent phospholipid transporter (Choi et al., 2019; Guissart et al., 2020; Heidari et al., 2021; Mikkelsen et al., 2019; Vestergaard et al., 2014). In this study, in order to further define the molecular basis for how mutations in *ATP8A2* cause CAMRQ4, we determined the expression, localization and functional activity of four additional ATP8A2 variants (Fig. 1A). Three (G447R, A772P and R1147W) have been genetically linked to CAMRQ4 (Mohamadian et al., 2020) (https://www.ncbi.nlm.nih.gov/clinvar) and one (E459Q) is a variant of uncertain significance. As part of this study, we used *in silico* programs to predict the effect of the mutations on the stability of the ATP8A2 variants and compared these results with the relative expression levels of the variants after solubilization in a mild detergent. Our results indicate that there is a strong correlation between the relative protein expression of the ATP8A2 variants and the respective protein stability predictions. *In silico* analysis was also extended to human disease-associated missense mutations reported for other P4-ATPases.

## RESULTS

### Location and sequence conservation of ATP8A2 variants

The general location of the various mutations in ATP8A2 implicated in CAMRQ4 is shown in Fig. 1A. In the present study, we investigated the molecular properties of variants harboring missense mutations in four residues. These residues included G447 and E459 in the N-domain, A772 in the P-domain, and R1147 in the C-terminal segment. The G447 residue is highly conserved in various vertebrate species and amongst all P4-ATPases except ATP9A and ATP9B (Fig. 1B). The A772 residue is highly conserved within the ATP8A and ATP8B families and several ATP10A members (ATP10A and ATP10D), and is also conserved in ATP8A2 from other vertebrates. The R1147 residue is also highly conserved in ATP8A and ATP8B P4-ATPases but is generally absent in other human P4-ATPase families, consistent with fact that the C-terminal segments of P4-ATPases differ widely in sequence. Finally, the E459 residue shares a high degree of conservation for ATP8A2 in most other vertebrates but is not well conserved in other P4-ATPases.

### Relative expression levels of ATP8A2 variants

**Fig. 2.**
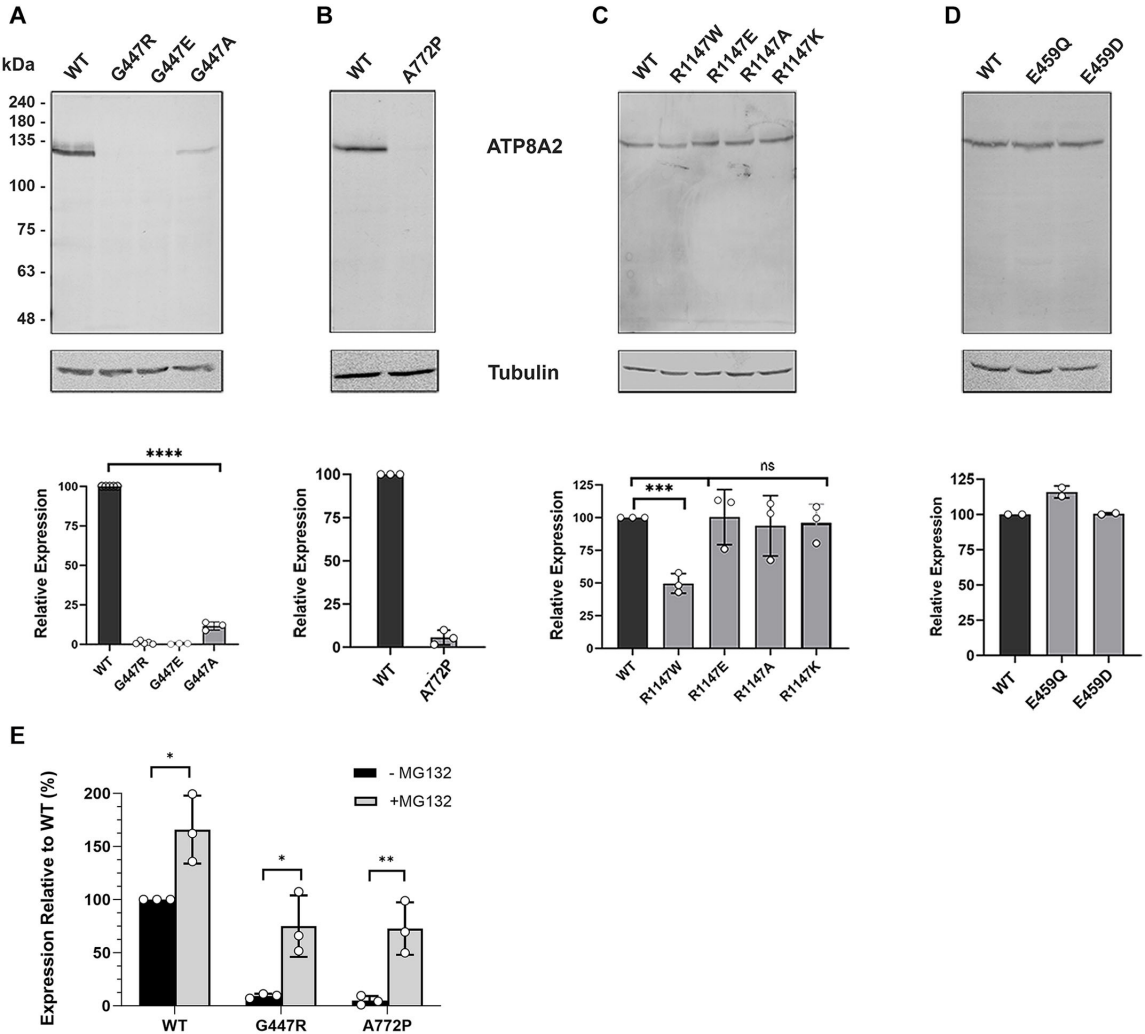
**Expression levels of wild-type ATP8A2 and variants implicated in CAMRQ4.** (A-E) Representative western blots (top) and quantification of protein levels (bottom) for independent experiments relative to WT ATP8A2. (A) Protein levels of wild-type (WT) ATP8A2 and (A) the G447R, G447E and G447A variants (****P<0.0001 for each construct); (B) the A772P variant; (C) the R1147W (***P=0.0003), R1147E, R1147A and R1147K variants (ns, not significant); and (D) the E459Q and E459D variants. Data are expressed as the mean±s.d. (E) Effect of the proteasome inhibitor MG132 on the expression of ATP8A2 variants. HEK293T cells transfected with the ATP8A2 variants were grown in the presence and absence of 10 µM MG132 for 5 h. Cell lysates were solubilized in SDS and analyzed for expression on western blots labeled for ATP8A2. Data are expressed as the mean±s.d. and shown as a percentage of WT ATP8A2 expression in the absence of MG132. WT ATP8A2, *P=0.0234; G447R, *P=0.0172; and A772P, **P=0.0094 The numbers of independent experiments are given by the circles. Statistical analysis was performed using paired two-tailed *t*-tests.

To investigate the molecular mechanisms by which mutations in these residues might cause CAMRQ4, we expressed wild-type (WT) or variant ATP8A2 with its accessory subunit CDC50A in HEK293T cells. After solubilization in 3-[(3-cholamidopropyl)dimethylammonio]-1-propanesulfate (CHAPS) detergent, the cell lysate was centrifuged at high speed to remove any misfolded protein aggregates and the protein levels of the ATP8A2 variants in the supernatant were compared to that of WT ATP8A2 by western blotting (Fig. 2A). The G447R variant, reported to be responsible for CAMRQ4 in an Iranian patient homozygous for this mutation (Mohamadian et al., 2020), showed no detectable levels relative to those of WT ATP8A2. The G447E and G447A variants were also studied to determine the effect of these substitutions on ATP8A2 expression. Similar to G447R, the G447E variant was undetectable on western blots labeled for ATP8A2, whereas the G447A variant was detectable at about 10% of WT ATP8A2 levels. These studies indicate that a glycine residue in this position is required for protein expression.

The A772P disease-associated variant (https://www.ncbi.nlm.nih.gov/clinvar) was also undetectable, indicating that the proline substitution causes significant protein misfolding (Fig. 2B). The R1147W variant was present at about 50% of WT ATP8A2 levels (Fig. 2C). Interestingly, ATP8A2 variants with a negatively charged glutamate residue (R1147E), a positively charged lysine residue (R1147K) or a neutral alanine residue (R1147A) yielded proteins that were detectable at WT levels. These data suggest that the bulky aromatic tryptophan residue is not well accommodated, leading to a reduction in expression. Finally, we examined the effect of the E459Q and E459D mutations on protein expression (Fig. 2D). Neither mutation affected ATP8A2 levels.

Previous studies have shown that the levels of highly misfolded ATP8A2 disease variants are markedly increased when the proteasome inhibitor MG132 is added to the transfected cells and the cell lysates are directly solubilized in SDS for analysis by western blotting (Choi et al., 2019; Guissart et al., 2020). To determine whether MG132 had a similar effect on the G447R and A772P variants, we incubated transfected HEK293T cells in the presence or absence of 10 µM MG132 for 5 h. The cell lysates were directly solubilized in SDS for analysis of ATP8A2 levels by western blotting (Fig. S1). The protein level of both variants significantly increased in the presence of MG132 (Fig. 2E), supporting the contention that these variants are highly misfolded and MG132 limits proteolytic degradation by the proteasome system of the cell.

### Immunofluorescence analysis of localization of ATP8A2 variants

**Fig. 3.**
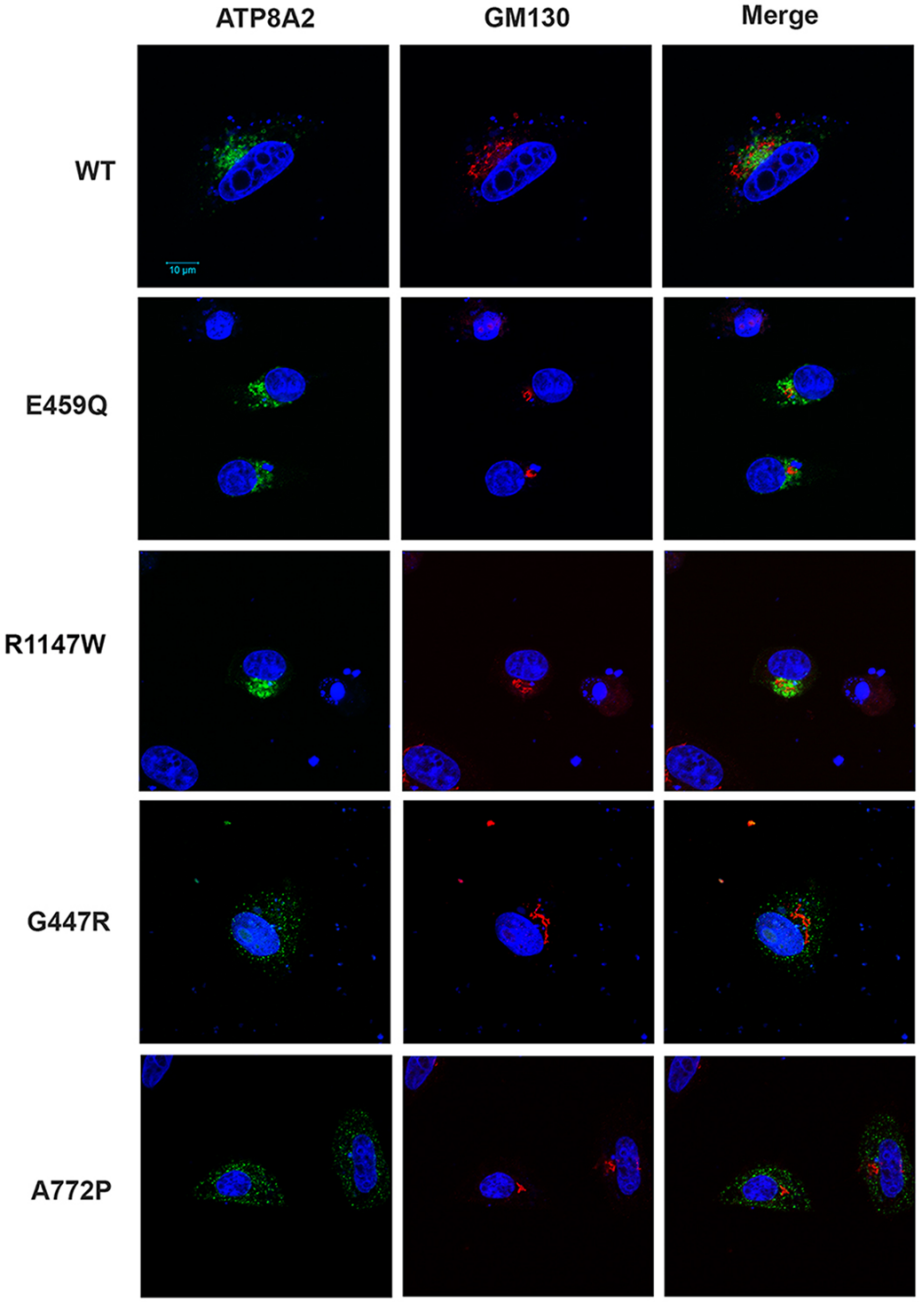
**Immunofluorescence micrographs of HeLa cells expressing WT ATP8A2-CDC50 complex or ATP8A2-CDC50A variants.** Cells were labeled for ATP8A2 with the Rho1D4 monoclonal antibody (green) and anti-GM130 polyclonal antibody (red) as a marker for the cis-Golgi and counterstained for nuclei with DAPI (blue). Images are representative of two independent experiments. Scale bar: 10 μm.

The effect of mutations on global protein folding can be evaluated by comparing the subcellular localization of the variant with that of the WT protein. Protein variants that fold in a native-like conformation can exit the ER and localize within cells in a manner similar to the WT protein, whereas globally misfolded proteins are typically retained in the ER by the quality control system of the cell and, in many cases, are rapidly degraded by the ER-associated protein degradation (ERAD) process. We visualized the subcellular localization of the ATP8A2 variants in HeLa cells co-transfected with the CDC50A construct using anti-GM130 (GOLGA2) as a marker for the Golgi. HeLa cells were used in place of HEK293T cells as they are larger and therefore more appropriate for immunofluorescence imaging. Previous studies have shown that a significant fraction of ATP8A2 localizes to Golgi–endosomes in various cultured cells, with a smaller fraction detected at the cell surface (Choi et al., 2019; Lee et al., 2015; van der Velden et al., 2010b). Segawa et al. (2016) have observed ATP8A2 in the plasma membrane of the mouse W3 cell line stably expressing the ATP8A2-CDC50A complex, together with intracellular staining reminiscent of Golgi–endosome staining. As shown in Fig. 3, WT ATP8A2 was observed to partially colocalize with the cis-Golgi marker GM130 in HeLa cells. The E459Q and R1147W variants displayed a similar subcellular localization. In the case of the G447R and A772P variants, however, only a small number of labeled cells were detected by immunofluorescence microscopy, consistent with the low levels of these variants observed by western blotting. The labeled cells showed small fluorescent punctate deposits. Lack of colocalization of these variants with the lysosome marker LAMP2 indicated that these variants were not associated with lysosomes (Fig. S2).

### PS activated ATPase activity

**Fig. 4.**
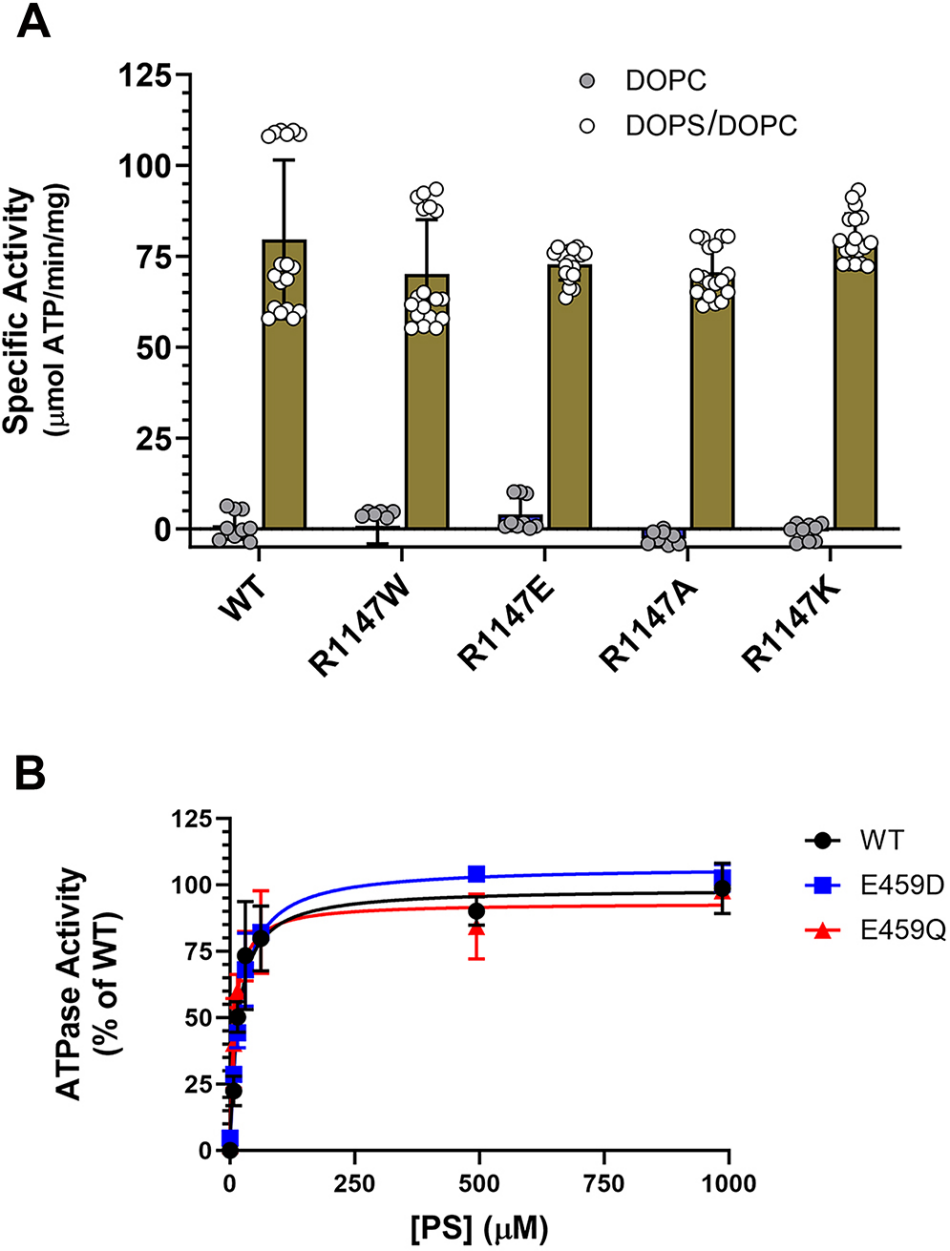
**ATPase activity of ATP8A2 variants in the presence of phosphatidylcholine (DOPC) or a mixture of DOPC and phosphatidylserine (DOPS).** (A) Specific activity of WT ATP8A2 and R1147W, R1147A and R1147K variants expressed as the mean±s.d. Circles indicate the number of independent experiments. (B) ATPase activity as a function of increase in phosphatidylserine (PS) for WT ATP8A2 and the E459D and E459Q variants. Data are from two independent experiments.

The effect of missense mutations on the function of ATP8A2 variants was determined by measuring their PS-dependent ATPase activity. Fig. 4A shows the specific ATPase activity of the R1147 variants in the presence of 1,2-dioleoyl-sn-glycero-3-phosphocholine (DOPC) or a mixture of 1,2-dioleoyl-sn-glycero-3-phospho-L-serine (DOPS) and DOPC (DOPS/DOPC). As in the case of WT ATP8A2, no significant ATPase activity was observed in the presence of DOPC alone. All R1147 variants, however, exhibited strong ATPase activity in the presence of 1 mM DOPS with a maximum reaction velocity (V_max_) in the range of 70-80 µmoles ATP hydrolyzed per minute per milligram of protein (µmol ATP/min/mg), similar to that of WT ATP8A2. The ATPase activity of the E459 variants as a function of increasing DOPS was also measured (Fig. 4B). Similar saturation kinetics were observed for human WT and the E459 ATP8A2 variants. The PS activation constant (K_a_) and V_max_ for WT ATP8A2 in the presence of DOPS yielded a K_a_ of 14.3±4.3 µM DOPS and V_max_ of 97.9±6.8 µmol ATP/min/mg, respectively (indicated as mean±s.d.), similar to values previously reported for human ATP8A2 (Choi et al., 2019). The E459Q and E459D variants were broadly similar, with a K_a_ of 8.4±3.1 µM DOPS and a V_max_ of 93.7±6.8 µmol ATP/min/mg for the E459Q variant, and a K_a_ of 19.3±4.2 µM DOPS and a V_max_ of 105.9±5.7 µmol ATP/min/mg for the E459D variant. The activities of the G447R and A772P variants were not determined owing to their low expression.

### Mapping the disease variants within the AlphaFold2 structure of ATP8A2

**Fig. 5.**
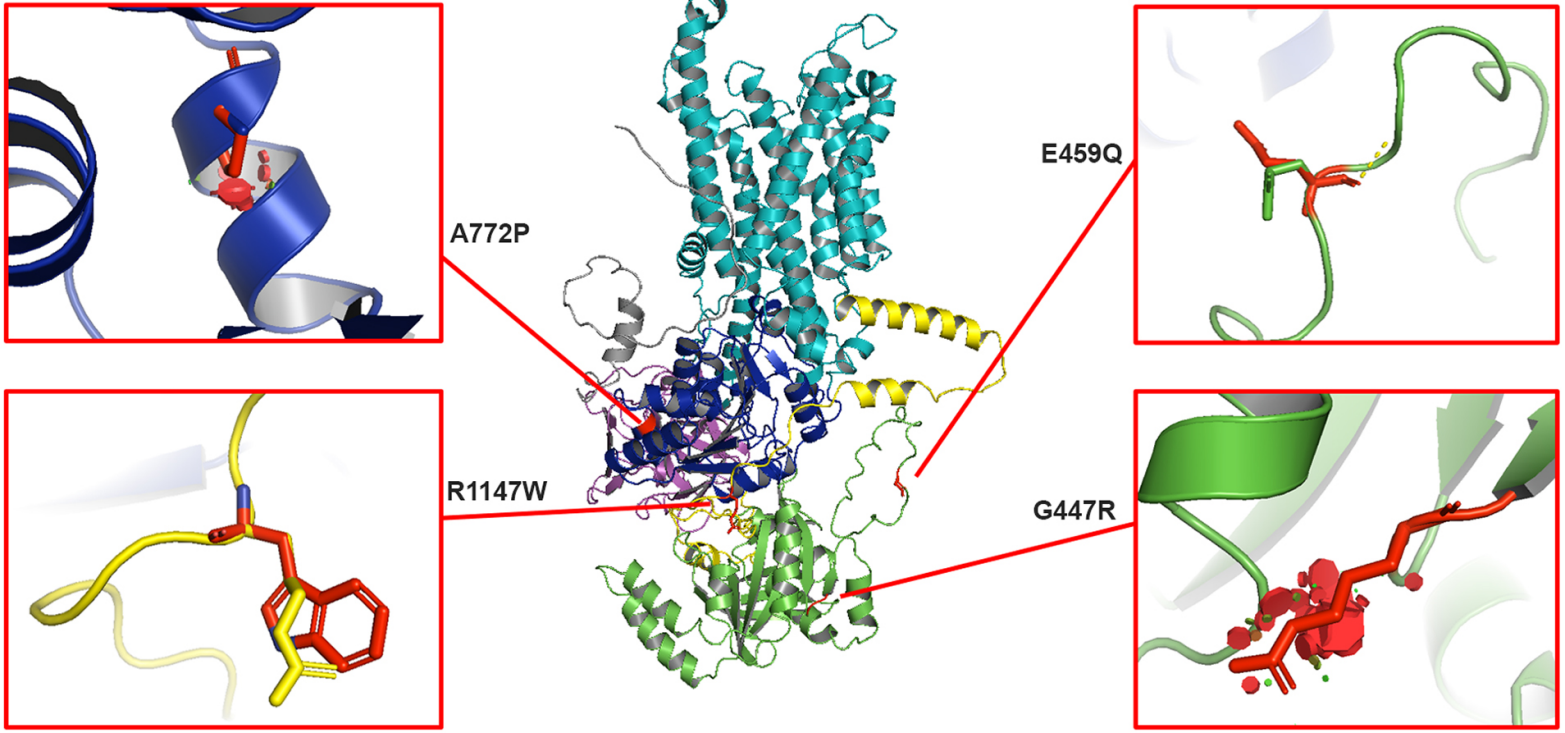
**The AlphaFold2 structure of ATP8A2.** The transmembrane domain (teal), phosphorylation domain (‘P’, blue), nucleotide-binding domain (‘N’, green) and actuator domain (purple) of ATP8A2 (α-subunit) are shown. The location of the four variants implicated in CAMRQ4 are shown. A772P is located within a helical structure of the Rossmann fold in the P-domain, R1147W is found within the C-terminal domain, E459Q is present in a flexible loop of the N-domain and G447R is located within the linker region within a β-sheet of the N-domain.

At present, the high-resolution structure of ATP8A2 has not been reported. However, the structure of ATP8A1, a P4-ATPase that is 67% identical in sequence to ATP8A2, has been determined in various states by cryo-electron microscopy (Hiraizumi et al., 2019). Alignment of the AlphaFold2 structure of ATP8A2 (Jumper et al., 2021) with the structure of ATP8A1 in its E2 state (PDB: 6K7L) indicates that the two structures are highly similar with a root mean square deviation of 1.171 Å (754 Cα atoms) (Fig. S3). We mapped the four variants implicated in CAMRQ4 to the AlphaFold2 structure to further evaluate how these substitutions may affect the structure and stability of the protein based on the spatial impact of the different side chain rotamers (Fig. 5).

The G447 residue is part of a β-turn within the N-domain. The replacement of this glycine with an arginine residue creates steric clashes in even the lowest-energy rotamer, likely leading to significant protein misfolding. The A772 residue is located within an α-helical segment of Rossmann fold of the P-domain. The Rossmann fold, a common characteristic of P-type ATPases, contributes to the binding of ATP. Substitution of this alanine with a proline results in the disruption of the α-helix and introduces a steric clash, which might affect the structure of the entire Rossman fold and result in global protein misfolding. The R1147 residue is located within a flexible region of the C-terminal segment of ATP8A2, upstream of the highly conserved Gly-Tyr-Ala-Phe-Ser (GYAFS) motif. This region was not resolved in the ATP8A1 structure, presumably due to the high flexibility of this segment. Substitution of the bulky tryptophan residue may lead to partial destabilization of the ATP8A2 structure and could cause a fraction of the protein to be in a misfolded state. Finally, the E459 residue is present a flexible loop of the N-domain. This position appears to be able to accommodate other residues including a glutamine and aspartic acid without significantly affecting the overall structure of ATP8A2.

### *In silico* protein stability predictions of ATP8A2 variants

A number of computational methods have been developed that predict the effect of an amino acid substitution on the stability of a protein. These programs typically use the three-dimensional structure and/or the sequence of the protein as an input together with other parameters such as solvent accessibility to predict free energy differences between the WT protein and the variant (Marabotti et al., 2021). We compared predictions for the stability of the ATP8A2 variants in the following programs: FoldX5 (Krieger and Vriend, 2014; Schymkowitz et al., 2005), INPS-3D (Savojardo et al., 2016), DUET (Pires et al., 2014) and DynaMut2 (Rodrigues et al., 2021) (Table 1). For these studies, we used the following free energy classification: stabilizing mutations (Gibbs free energy difference or ΔΔG<−0.5 kcal/mol), destabilizing mutations (ΔΔG>0.5 kcal/mol) and neutral mutations (−0.5 kcal/mol<ΔΔG<0.5 kcal/mol). All programs predicted the amino acid substitutions in G447 as destabilizing, but only FoldX5 predicted the A772P substitution as severely destabilizing. The E459 variants were predicted as neutral or mildly destabilizing. Some variability in predictions were observed for the R1147 variants but, in general, these were in the range of neutral to mildly destabilizing (Table 1).

**Table 1.**
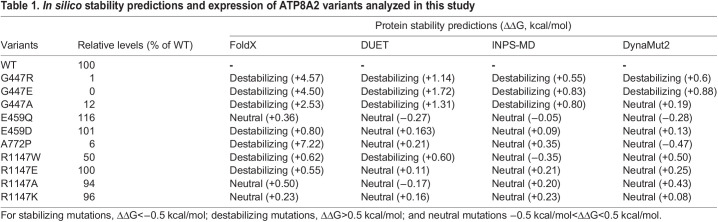
*In silico* stability predictions and expression of ATP8A2 variants analyzed in this study

Interestingly, the predictions were in general agreement with the protein expression studies carried out here (Table 1). The G447 and A772 variants predicted to be destabilizing correlated with their absent or low protein levels, and the E459 and most R1147 variants predicted to be neutral or mildly destabilizing displayed protein levels similar to those of WT ATP8A2. We note here that these studies do not generally reveal stabilizing mutations as the protein levels of the mutants were rarely greater than those of the WT protein.

We extended the *in silico* predictions to other ATP8A2 disease-associated variants for which expression data have been previously reported (Table 2). Nine variants that were predicted to be highly destabilizing by FoldX5 expressed at exceedingly low levels, consistent with severe protein misfolding, whereas the four variants that were predicted to be neutral or stabilizing expressed at WT levels. Comparison of the four programs indicate that FoldX5 had 100% correlation with expression for the ATP8A2 variants (Table 2), whereas the other three programs showed a 60-70% correlation. The ClinVar database (https://www.ncbi.nlm.nih.gov/clinvar/) shows a large number of missense mutations that may underly CAMRQ4 or related neurodevelopmental disorders. In Table S1, we further analyzed the effect of 11 of these mutations on the stability of ATP8A2 using three of these computational programs. Nine of these mutations were predicted to cause destabilization of ATP8A2 using FoldX5, whereas fewer mutations were predicted to be destabilizing by DUET and DynaMut2. Expression studies are not available for these ATP8A2 variants.

**Table 2.**
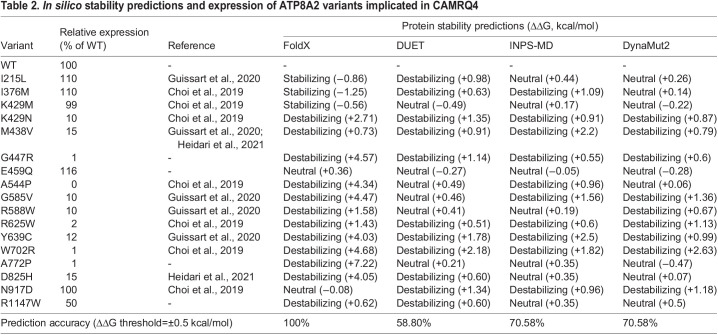
*In silico* stability predictions and expression of ATP8A2 variants implicated in CAMRQ4

### *In silico* analysis of ATP8B1, ATP11C and ATP11A disease variants

We expanded our analysis to other P4-ATPases associated with genetic diseases. ATP8B1 has been the most extensively studied owing to its association with both BRIC1 and PFIC1 (Bull et al., 1998; Klomp et al., 2004; Takatsu et al., 2014; van der Velden et al., 2010a). In Table S2, we analyzed 25 clinical variants. There was general agreement for the three programs with a few exceptions. Of the 11 variants linked to BRIC1, nine were considered destabilizing by most programs. One variant, D454G, linked to BRIC1 was predicted to have a stabilizing or neutral effect by all three programs. However, this variant expressed at low to moderate levels (Folmer et al., 2009b; van der Velden et al., 2010a). Seven of the 13 PFIC1 variants were predicted to be destabilizing, with six others predicted to be stabilizing. Of these mutations, five have been characterized in terms of their expression. FoldX5 and DUET showed the highest degree of agreement between the reduction in expression and destabilization of the mutations, whereas DynaMut2 showed a lower agreement. The effect of the mutations on ATP8B1 can be further assessed from the structure of ATP8B1. For example, substitution of G1040 within transmembrane segment 7 (M7) for an arginine residue (G1040R) appears to destabilize ATP8B1 as a result of steric clashes and introduction of a charged side chain in a hydrophobic environment (Fig. S4). Likewise, the G308V mutation also introduces a bulky side chain within a β-structure of the actuator domain, introducing steric clashes. The I661T mutation, in contrast, likely destabilizes ATP8B1 by introducing the polar threonine side chain into a region occupied by hydrophobic residues. Interestingly, the disease-associated L127P mutation in transmembrane segment 1 (M1), which was predicted to destabilize ATP8B1 by two of the three programs, mildly reduced expression levels in one report (Takatsu et al., 2014). In this case, it is likely that this substitution introduces a kink in the helical structure, which only marginally affects the protein folding. The L127P mutation, however, abolished phosphatidylcholine transport activity in ATP8B1 (Takatsu et al., 2014), and significantly reduced expression and ATPase activity have been reported for the equivalent mutation (I91P) in ATP8A2 (Gantzel et al., 2017).

ATP11C and ATP11A have also been linked to inherited diseases (Arashiki et al., 2016; Pater et al., 2022; Segawa et al., 2021; van Dijk et al., 2023). To date, 11 missense mutations in ATP11C have been implicated in congenital hemolytic anemia. Of these mutations, five were predicted to be destabilizing by most of the programs, whereas the remaining six were predicted to be either neutral or stabilizing (Table S3). The T418N and L789F ATP11C variants are the only ones that have been characterized at a molecular level (Arashiki et al., 2016; Liou et al., 2019; van Dijk et al., 2023). These mutations reduced expression levels of ATP11C, consistent with the destabilizing effects of these mutations predicted by all three programs.

Three disease-associated variants have been identified in ATP11A. S4N is associated with hearing loss, whereas A37V and Q84E are associated with leukodystrophy and hypomyelination (Table S3). These three mutations are in close proximity to the N-terminus of the protein and were not predicted by a majority of the programs to destabilize the protein.

## DISCUSSION

In this study, we examined the subcellular localization, expression and functional properties of four ATP8A2 mutants that have been implicated in CAMRQ4. Two variants, G447A and A772P, were undetectable in cell lysates of transfected cells by western blotting after solubilization in CHAPS buffer and failed to localize to the Golgi–recycling endosomes in the few labeled cells observed by immunofluorescence microscopy. These results indicate that these mutations cause CAMRQ4 through a mechanism involving severe protein misfolding. This is further supported in a structural model of ATP8A2 that shows that the G447 and A772 residues are located in highly structured regions within the N-domain and P-domain, respectively, such that the G447E and A772P substitutions cause significant structural defects and steric clashes. In contrast, the E459Q variant exhibited protein levels and subcellular localization similar to those of WT ATP8A2. Furthermore, this variant displayed PS-activated ATPase activity similar to that of WT ATP8A2. This suggests that the E459Q mutation may not cause CAMRQ4. The R1147W variant expressed at about half of WT levels. This variant is likely pathogenic, but individuals homozygous for this mutation may display a milder phenotype owing to its moderate expression and residual activity. A more complete genetic and clinical evaluation of patients with this mutation would be of interest.

Protein stability is commonly determined by measuring the transition between the folded and unfolded states upon the exposure to changes in temperature, pH or denaturant (Atsavapranee et al., 2021; Chib et al., 2015; Crowther et al., 2010; Murayama and Tomida, 2004). However, such studies are difficult to carry out for membrane proteins as these proteins are generally difficult to produce and purify in sufficient quantities for such measurements, and evaluating the transition from the folded to unfolded state, often performed using fluorescence dyes, is difficult to carry out and interpret owing to the hydrophobic nature of these proteins. We reasoned that the level of membrane protein expression after solubilization in a mild detergent may provide an alternative way to assess the effect of mutations on the stability of membrane proteins. Unstable, misfolded membrane proteins are typically retained within the ER where they can undergo aggregation and/or degradation by the ERAD pathways of the cell. Any residual misfolded proteins typically aggregate in mild detergent such that they can be removed by centrifugation. The supernatant can then be analyzed for protein expression by western blotting for comparison with the WT protein. We compared the level of expression of ATP8A2 variants using four commonly used software programs designed to predict Gibbs free energy differences (ΔΔG) between the folded and unfolded states of the WT and mutant protein. Our studies indicate that there is considerable agreement between expression and stability predictions for the ATP8A2 variants studied here and those for which expression data are available. An illustrative example of this is apparent for two different mutations (K429N and K429M) in the same residue of ATP8A2 found in patients with CAMRQ4 (Guissart et al., 2020; Martin-Hernandez et al., 2016; McMillan et al., 2018). The K429N variant has been reported to express at exceedingly low levels, whereas the K429M variant is expressed at WT levels (Choi et al., 2019). All programs predicted K429N as a destabilizing mutation and K429M as a neutral or stabilizing mutation, consistent with the expression profile. However, functional studies indicate that both variants are devoid of ATPase activity, in line with the key role of K429 within the conserved Asp-Lys-Thr-Gly-Thr (DKTGT) motif required for transient phosphorylation as part of the reaction cycle. Of the programs used in our study, FoldX5 showed a particularly high degree of agreement with the *in vitro* expression data. This program calculates the difference in free energy based on several variables, including Van der Waals force contributions of all atoms, comparisons of intramolecular to intermolecular hydrogen bonding, electrostatic contributions of charged groups, differences in solvation energy in the folded and unfolded state, as well as entropic costs of fixing and maintaining structural conformations.

Most disease-associated ATP8A2 variants that have been studied at a molecular level result in a loss of function through a mechanism involving global protein misfolding, resulting in low detectable protein levels. Of the 17 ATP8A2 disease-associated variants studied (Tables 1 and 2), 12 displayed very low expression. Most of the other variants are expressed at normal levels and are predicted to be stable. These variants, however, generally display little or no PS-activated ATPase activity as a result of the loss in a key residue required for the ATP-dependent transport activity of ATP8A2 (Choi et al., 2019; Heidari et al., 2021; McMillan et al., 2018; Mikkelsen et al., 2019; Vestergaard et al., 2014). In such cases, expression and *in silico* analyses are not sufficient to identify these mutations as pathogenic without additional information on how these mutations affect the functional activity of the protein.

Insight into the stability of disease-associated variants is of interest for the development of therapeutic strategies. It has been previously shown that compounds such as 4-phenyl butyrate and MG132 enhance the expression levels of misfolded ATP8B1 and ATP8A2 disease variants (Choi et al., 2019; Heidari et al., 2021; van der Velden et al., 2010a). Application of these or other drugs that improve protein folding and/or reduce degradation could partly rescue the disease phenotype in animal models and ultimately lead to clinical trials.

## MATERIALS AND METHODS

### Reagents

DOPC and DOPS were purchased from Avanti Polar Lipids (Alabaster, AL, USA). ATP and ascorbic acid were purchased from Sigma-Aldrich, CHAPS from Anatrace (Maumee, OH, USA), ProteaseARREST protease inhibitor cocktail from G-Biosciences (St. Louis, MO, USA) and 1D4 peptide from Biomatik (Kitchener, ON, USA). MG132 was from ApexBio (Houston, TX, USA). HEK293T and HeLa cells were obtained from American Type Culture Collection through Cedarlane Laboratories (Burlington, Ontario, Canada). The Rho1D4 antibody initially produced in house (Hodges et al., 1988; Molday and Molday, 2014) was obtained from the University of British Columbia (https://uilo.ubc.ca/industry-partners/access-ubc-technologies). The primary antibody against tubulin was from Abcam (ab15568; Toronto, Ontario, Canada) and the antibody against LAMP2 was from Santa Cruz Biotechnology (sc-5571; Dallas, TX, USA). Primary antibodies against ATP8A2 (Atp6C11 and Cdc50-7F4) have been described previously (Coleman et al., 2009; Coleman and Molday, 2011). Restriction enzymes, T4 DNA ligase, Antarctic Phosphatase and Phusion polymerase were acquired from New England Biolabs (Whitby, Ontario, Canada). Primers used for mutagenesis were ordered from Integrated DNA Technologies (Coralville, IA, USA).

### Plasmid construct design

The human *ATP8A2* construct (NM_016529.6) was previously developed in a pcDNA3.1 plasmid (Invitrogen, V790-20) engineered to contain a C-terminal 1D4 tag (Coleman et al., 2009; Lee et al., 2015). Mutant constructs were developed using site-directed mutagenesis with overlapping primers designed to incorporate each mutation into the WT ATP8A2 construct using Phusion polymerase. Mutations were verified by Sanger sequencing and re-ligated into the WT plasmid using the Kpn1 and Xba1 restriction enzymes. The human *CDC50A* construct (NM_018247.4) was cloned into a pcDNA3.1 plasmid using the Kpn1 and Not1 restriction enzymes.

### Cell culture and protein expression

HEK293T cells were cultured in Dulbecco's modified Eagle medium (Sigma-Aldrich) supplemented with 8% bovine growth serum (Thermo Fisher Scientific), 100 IU/ml penicillin, 100 μg/ml streptomycin and 2 mM L-glutamine. The cells grown on 10 cm plates to ∼30% confluency were co-transfected with 5 μg each of the ATP8A2 plasmid and CDC50A plasmids and 30 μg of polyethylenimine (PEI; Sigma-Aldrich, P3143) per plate. The cells were harvested after 48 h and pelleted at 1500 ***g*** (Sorval Legend RT). The cells were resuspended in lysis buffer (150 mM NaCl, 50 mM HEPES/NaOH pH 7.5, 5 mM MgCl_2_, 20 mM CHAPS, 1× ProteaseARREST, 1 mM dithiothreitol and 0.5 mg/ml DOPC) with stirring at 4°C. After 30 min, the detergent-insoluble membrane fraction was removed from the detergent-soluble fraction by centrifugation at 40,000 ***g*** (TLA 55 rotor, 03U 692, Beckman Coulter) for 12 min. The solubilized ATP8A2–CDC50A proteins were then used for downstream applications. Protein expression was measured by western blotting analysis and normalized to a tubulin loading control as previously described (Choi et al., 2019).

### MG132 proteasome inhibition

HEK293T cells were co-transfected with the given ATP8A2 and CDC50A constructs at ∼80% confluency and grown for 24 h. The medium was replaced with fresh medium. After 15 min, the cells were incubated with either a DMSO control (1/1000 dilution) or 10 µM MG132 (ApexBio, A2585) for 5 h, at which point the cells were resuspended in lysis buffer containing 4% SDS as a substitute for CHAPS detergent with stirring at room temperature. The sample was centrifuged at 40,000 ***g*** for 12 min and the supernatant was used for analysis of protein levels by western blotting. The protein levels were quantified and expressed as a percentage of WT protein levels without MG132 and normalized to those of a housekeeping protein (tubulin). Paired two-tailed *t*-tests were used to determine significance.

### Immunoaffinity protein purification

Purification of the ATP8A2–CDC50A complex was achieved using immunoaffinity chromatography as previously described (Coleman et al., 2009; Coleman and Molday, 2011). Anti-Rho1D4 columns were prepared through the coupling of the Rho1D4 monoclonal antibody to CNBr-activated Sepharose 2B beads (Pharmacia, 17-0140-01) as described previously (Molday and Molday, 2014). The detergent-solubilized cell lysate was incubated with the Rho1D4 immunoaffinity matrix in spin columns for 45 min at 4°C. The supernatant was then removed, and the spin columns were washed six times with 400 µl of column wash buffer (150 mM NaCl, 50 mM HEPES/NaOH pH 7.5, 5 mM MgCl_2_, 10 mM CHAPS, 1 mM dithiothreitol, 0.5 mg/ml DOPC). The bound protein was eluted from the column by two additions of 50 µl of wash buffer containing 0.5 mg/ml of the 1D4 peptide. Purified protein concentration was determined by SDS-PAGE, visualized with Coomassie blue staining and quantified with bovine serum albumin (BSA) standards.

### ATPase assay

The ATPase activity of purified ATP8A2 constructs was assayed using a previously described colorimetric method (Chifflet et al., 1988; Coleman et al., 2009). Titration curves for the velocity of ATP8A2–CDC50A-driven ATP hydrolysis were determined as a function of substrate concentration. To measure the ATPase velocity as a function of lipid substrate, 1 ng of purified protein was incubated with 5 mM ATP and 2.5 mg/ml of different ratios of DOPC:DOPS. The ATP and lipid samples were prepared in ATPase assay buffer (50 mM HEPES/NaOH, pH 7.5, 150 mM NaCl, 12.5 mM MgCl_2_, 1 mM dithiothreitol and 10 mM CHAPS). The mixture (25 µl) was incubated at 37°C for 30 min. The activity of the protein was terminated by the addition of 25 µl of 12% SDS. Free phosphate was measured by the addition of 75 µl of solution 1 (6% ascorbic acid, 1% ammonium molybdate in 1 N HCl) for 5 min, followed by 120 μl of solution 2 (2% sodium citrate, 2% sodium meta-arsenite, 2% acetic acid). The reaction resulted in a color shift, allowing for the measurement of free phosphate absorbance at 850 nm in a microplate reader. These values were compared to those of a phosphate standard curve, allowing for calculation of the reaction velocity (µmoles of inorganic phosphate released per minute per milligram of protein) for each data point in the given assay. Each measurement was performed in technical triplicates. The data was then fitted to a Michaelis–Menten equation using GraphPad Prism 9. Maximum reaction velocity (V_max_) and PS activation constant (K_a_) were then calculated. Technical triplicates were performed for two or more independent experiments involving distinct protein preparations.

### SDS-PAGE and western blotting

Proteins were separated by SDS gel electrophoresis on 9% polyacrylamide gels and either stained with Coomassie blue or transferred to PVDF membranes (Millipore, Bedford, MA, USA) and blocked with 1% milk in PBS for 45 min as previously described (Choi et al., 2019). The blots were labeled with hybridoma cell culture fluid containing either the Rho1D4 (diluted 1:100 in PBS) or Cdc50-7F4 (diluted 1:12 in PBS) monoclonal antibodies. The blots were also labeled with anti-tubulin antibody (1:3000) serving as a loading control. After 1 h, the gels were washed stringently with PBS containing 0.05% Tween 20 (PBST), incubated for 40 min with secondary antibody (goat anti-mouse conjugated with IR dye 680 or donkey anti-rabbit conjugated with IR dye 800; 926-68070 and 926-32213; LI-COR, Lincoln, NE, USA) diluted 1:20,000 in PBST containing 0.5% milk. After washing in PBST, the blots were imaged on a LI-COR Odyssey infrared imaging system.

### Immunofluorescence microscopy

HeLa cells grown on glass coverslips in six-well tissue culture plates were co-transfected at 50% confluence with 1.25 µg of *ATP8A2-1D4* and 1.25 µg of *CDC50A* plasmids using PEI as a transfection agent. After 48 h, the cells were washed in PBS and fixed with 4% paraformaldehyde for 15 min, then blocked and permeabilized for 1 h with 10% normal goat serum (NGS; Sigma-Aldrich, NS02L) and 0.2% Triton X-100. The cells were treated for 2 h with primary antibody diluted in 100 mM phosphate buffer (pH 7.4) containing 2.5% NGS and 0.05% Triton X-100. The primary antibodies were Rho1D4 hybridoma culture fluid (1:50 dilution), rabbit anti-GM130 antibody (1:2000, SAB5700801, Sigma-Aldrich) for labeling the cis-Golgi and anti-LAMP2 (1:100) for labeling lysosomes. The coverslips were washed and labeled with goat anti-mouse immunoglobulin tagged with Alexa Fluor 488 (A11029, Thermo Fisher Scientific) and goat anti-rabbit immunoglobulin tagged with Alexa Fluor 594 (A11012, Thermo Fisher Scientific) for 0.5 h and stained with the nuclear dye 4′,6-diamidino-2-phenylindole (DAPI). Coverslips were washed and mounted with Mowiol medium (Sigma-Aldrich, 32459-0) on glass slides prior to imaging. The reagents for fixing, permeabilizing and labeling were prepared in 100 mM phosphate buffer (pH 7.4). The secondary antibodies were used at 1:1000 dilution. Fluorescence images were acquired using a 63× oil immersion objective on a Zeiss LSM 700 confocal microscope equipped with Zen Image analysis software. Composite figures were prepared using Photoshop software.

### *In silico* stability measurements

The stability of WT and ATP8A2 variants were predicted using the following software programs: FoldX5 (https://foldxsuite.crg.eu/), DUET (Pires et al., 2014), INPS-3D (Savojardo et al., 2016) and DynaMut2 (Rodrigues et al., 2021). The protein structures were generated in AlphaFold2 (Jumper et al., 2021) and were repaired using the FoldX5 plugin with the YASARA viewer (http://www.yasara.org/). The Gibbs free energy change was calculated and reported as ΔΔG_mutant_−ΔΔG_WT_. PyMOL Molecular Graphics System (version 1.7, Schrodinger) was used to display structures and assess the impact of amino acid substitutions on the structure.

## Supplementary Material

10.1242/dmm.050546_sup1 Supplementary information
