# Supplementary material for: Functional and in silico analysis of ATP8A2 and other P4-ATPase variants associated with human genetic diseases
Source: Dis Model Mech. 2024 Apr 24;17(6):dmm050546. doi: 10.1242/dmm.050546 (PMC11073571; doi:10.1242/dmm.050546)
Supplement: Supplementary information [file dmm-17-050546-s1.pdf]

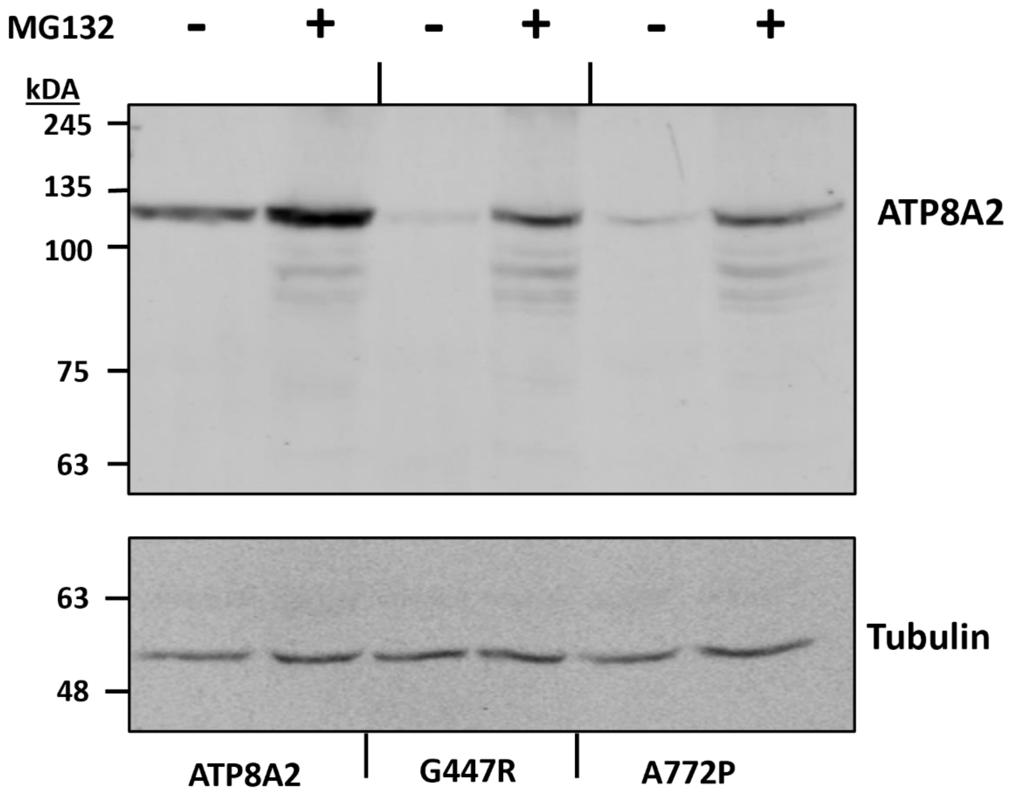

Fig. S1. Effect of MG132 proteosome inhibitor on the expression levels of ATP8A2. ATP8A2 variants were expressed in HEK293T cells in the presence and absence of 10 µM MG132 for 5 h. Cell lysates were solubilized in SDS and analyzed on western blots labeled for ATP8A2 with the Rho 1D4 antibody. Tubulin was used as a loading control.

Disease Models & Mechanisms • Supplementary information

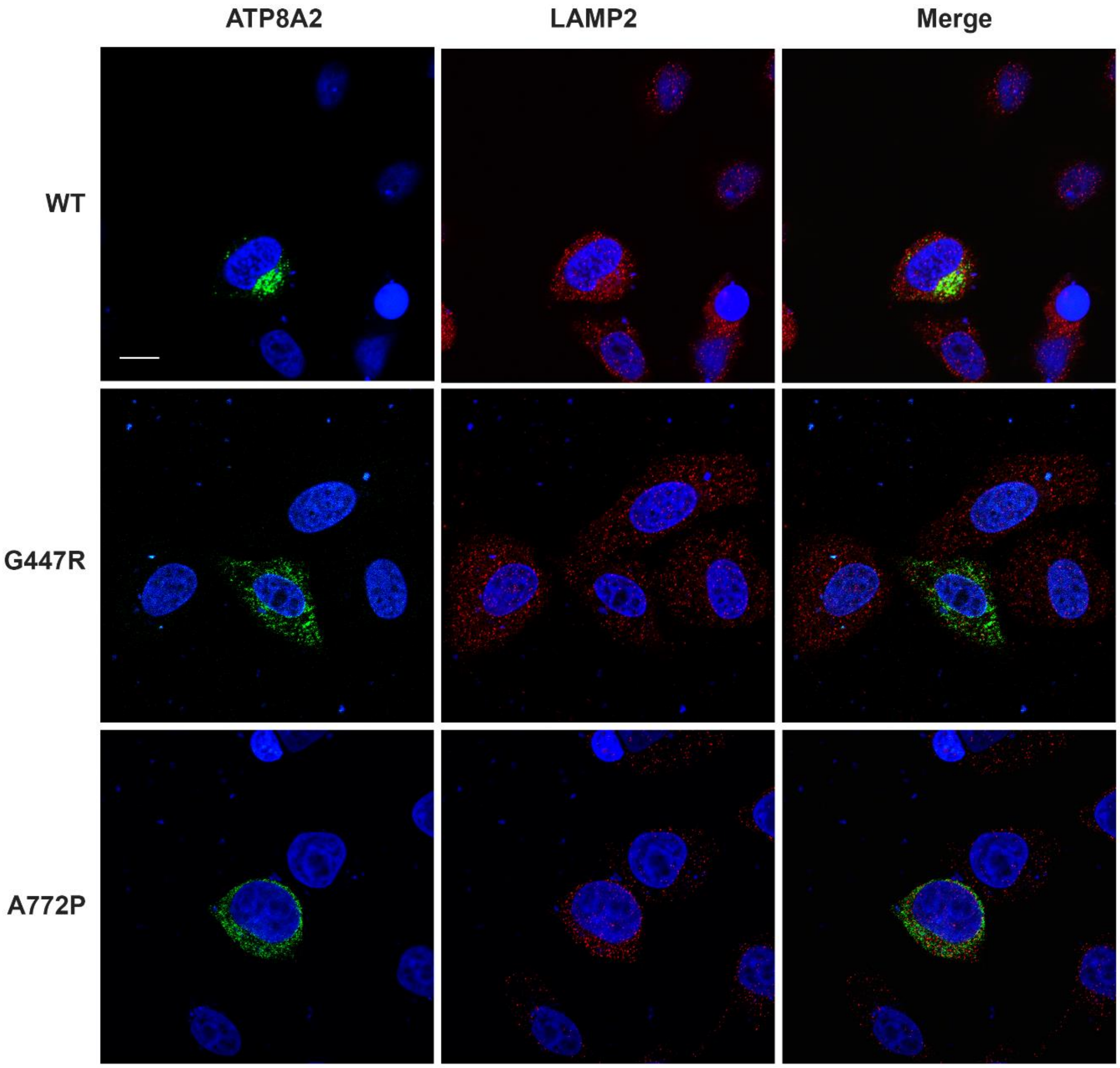

Fig. S2. Immunofluorescence micrograph of HELA cells labeled for ATP8A2 variants and lysosomes. Cells co-transfected with wild-type (WT) ATP8A2 or variants (G447R or A772P) and CDC50A were labeled for ATP8A2 with the Rho 1D4 monoclonal antibody to the C-terminal tag (Green) and a polyclonal antibody to LAMP2 as a lysosome marker (Red) and counterstained for nuclei with DAPI (blue). Bar – 10 µM.

Disease Models & Mechanisms • Supplementary information

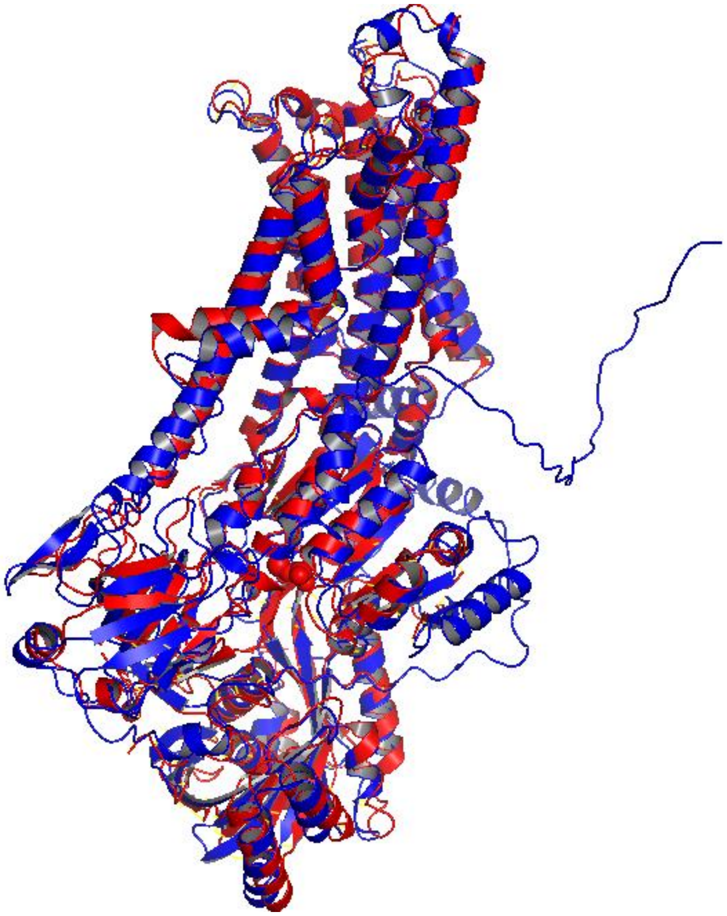

Fig. S3. Alignment between the Cryo-EM structure of ATP8A1 (blue) (6K7L) and the αFold structure of ATP8A2 (red) (Q9NTI2). The RMSD was calculated to be 1.171 Å.

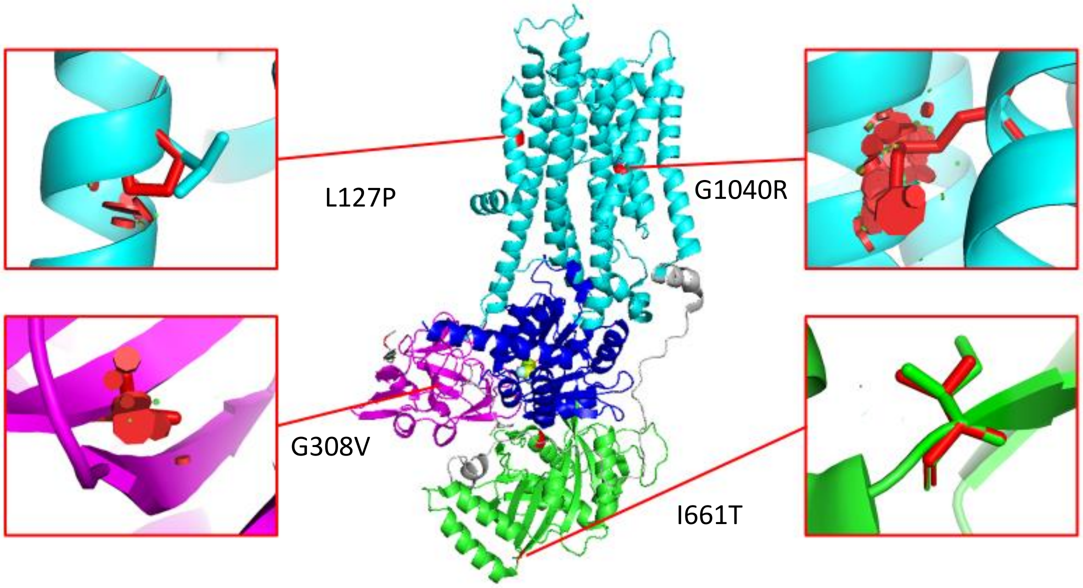

Fig. S4. The location of four missense mutations observed in ATP8B1 implicated in progressive familial intrahepatic cholestasis (PIFC1). The cryo-EM structure of ATP8B1 (7PY4) in the E2 conformation was visualized using Pymol. L127P and G1040R are located within the transmembrane domains of the flippase. G308V is located within a beta-sheet secondary structure of the actuator domain. I661T is within a beta-sheet secondary structure of the nucleotide binding domain. Steric clashing is indicated by the red discs shown with size being proportional to degree of clashing.

Disease Models & Mechanisms • Supplementary information

Table S1. In-silico predictions for uncharacterized ATP8A2 variants associated with CAMRQ4.

	Protein Stability Prediction		
**Variants**	**FoldX (ΔΔGStability) (Kcal/mol)**	**DUET (ΔΔGStability) (Kcal/mol)**	**DynaMut2 (ΔΔGStability) (Kcal/mol)**
A53V	Neutral (+0.13)	Neutral (-0.03)	Destabilizing (+1.14)
Y248C	Destabilizing (+1.56)	Neutral (-0.40)	Destabilizing (+0.63)
P492A	Destabilizing (+1.21)	Neutral (-0.06)	Neutral (-0.11)
M555I	Destabilizing (+2.36)	Stabilizing (-0.54)	Neutral (+0.25)
C628Y	Destabilizing (+1.91)	Destabilizing (+0.96)	Destabilizing (+0.54)
K644T	Neutral (-0.02)	Destabilizing (+0.62)	Neutral (+0.32)
R659Q	Destabilizing (+1.00)	Destabilizing (+0.75)	Neutral (+0.10)
V687F	Destabilizing (+17.04)	Destabilizing (+1.50)	Destabilizing (+1.16)
R778Q	Destabilizing (+1.69)	Destabilizing (+0.78)	Destabilizing (+0.63)
A897T	Destabilizing (+1.17)	Destabilizing (+0.88)	Neutral (-0.18)
I966V	Destabilizing (+0.57)	Neutral (+0.45)	Neutral (+0.01)

Disease Models & Mechanisms • Supplementary information

Table S2. Comparison of biochemical and in-silico characterization for disease associated variants of ATP8B1.

Condition(s)	ATP8B1 Disease Variants	Expression (Relative to WT)	Ref.	Protein Stability Predictions: FoldX (ΔΔGStability) (Kcal/mol)	DUET (ΔΔGStability) (Kcal/mol)	DynaMut2 (ΔΔGStability) (Kcal/mol)
BRIC1/ICP	D70N	Reduced<br>WT-Like	1,2	Stabilizing (-0.58)	Destabilizing (+0.86)	Destabilizing (+0.75)
PFIC1	L127P	Reduced*<br>WT-like	1,3	Destabilizing (+4.30)	Destabilizing (+1.91)	Neutral (+0.33)
PFIC1	L288S	Reduced*	1	Destabilizing (+6.11)	Destabilizing (+3.60)	Destabilizing (+3.29)
BRIC1	G308D	-	-	Destabilizing (+4.39)	Destabilizing (+2.31)	Destabilizing (+1.81)
PFIC1	G308V	Reduced*	1,2,3	Destabilizing (+3.52)	Stabilizing (-0.73)	Neutral (+0.07)
BRIC1	I344F	WT-Like	1	Destabilizing (+6.96)	Destabilizing (+1.63)	Destabilizing (+1.48)
PFIC1	S403Y	-	-	Stabilizing (-1.91)	Neutral (-0.18)	Destabilizing (+0.74)
PFIC1	R412P	-	-	Destabilizing (+8.06)	Destabilizing (+1.36)	Destabilizing (+0.52)
BRIC1	S453Y	-	-	Destabilizing (+13.10)	Destabilizing (+0.57)	Destabilizing (+1.01)
BRIC1	D454G	Reduced*	1,3	Stabilizing (-2.09)	Neutral (-0.18)	Neutral (-0.27)
PFIC1	T456M	-	-	Neutral (-0.06)	Stabilizing (-0.79)	Stabilizing (-1.25)
PFIC1	Y500H	-	-	Destabilizing (+1.32)	Destabilizing (+0.35)	Neutral (-0.40)
PFIC1	H535L	-	-	Destabilizing (+1.90)	Destabilizing (+0.48)	Neutral (+0.07)
PFIC1	D554N	Reduced*	2,3	Destabilizing (+0.66)	Destabilizing (+0.88)	Destabilizing (+0.98)
BRIC1	R600W	-	-	Destabilizing (+1.61)	Destabilizing (+0.68)	Destabilizing (+0.99)
BRIC1	R600Q	-	-	Destabilizing (+1.89)	Destabilizing (+1.69)	Destabilizing (+0.65)
BRIC1	R628W	-	-	Destabilizing (+0.74)	Destabilizing (+0.61)	Neutral (+0.26)
BRIC1/PFIC1	I661T	Reduced*	1,2,3	Destabilizing (+3.70)	Destabilizing (+2.95)	Destabilizing (+2.52)
PFIC1	D688G	-	-	Destabilizing (+1.45)	Destabilizing (+0.98)	Destabilizing (+0.61)
BRIC1	I694T	-	-	Destabilizing (+3.65)	Destabilizing (+2.85)	Destabilizing (+2.37)
PFIC1	G733R	-	-	Stabilizing (-0.59)	Neutral (+0.25)	Neutral (+0.49)
PFIC1	F853S	-	-	Destabilizing (+5.79)	Destabilizing (+2.87)	Destabilizing (+2.74)
ICP	R867C	Reduced*	2	Neutral (+0.36)	Destabilizing (+0.81)	Neutral (+0.05)
BRIC1/PFIC1	G892R	-	-	Destabilizing (+28.84)	Destabilizing (+0.93)	Destabilizing (+0.71)
PFIC1	G1040R	Reduced*	1,2,3	Destabilizing (+18.86)	Destabilizing (+1.25)	Destabilizing (+0.58)

*Reduced expression is defined as being ~<70% of WT protein expression levels

Table S3. Comparison of biochemical and in-silico characterization for disease associated variants of ATP11C and ATP11A.

Condition(s)	Disease Variants	Expression (Relative to WT)	Ref.	Protein Stability Predictions: FoldX (ΔΔGStability) (Kcal/mol)	DUET (ΔΔGStability) (Kcal/mol)	DynaMut2 (ΔΔGStability) (Kcal/mol)
	**ATP11C**					
Congenital Hemolytic Anemia	R335Q	-	-	Destabilizing (+1.03)	Neutral (+0.08)	Destabilizing (+0.55)
Congenital Hemolytic Anemia	K370R	-	-	Neutral (-0.43)	Neutral (+0.35)	Destabilizing (+0.55)
Congenital Hemolytic Anemia	E390D	-	-	Neutral (-0.07)	Destabilizing (+0.54)	Neutral (+0.45)
Congenital Hemolytic Anemia	T418N	Reduced	4	Destabilizing (+2.99)	Destabilizing (+1.00)	Destabilizing (+0.68)
Congenital Hemolytic Anemia	I723R	-	-	Destabilizing (+1.23)	Destabilizing (+0.92)	Destabilizing (+0.59)
Congenital Hemolytic Anemia	V788L	-	-	Neutral (-0.44)	Neutral (+0.30)	Destabilizing (+0.64)
Congenital Hemolytic Anemia	L789F	Reduced	5	Destabilizing (+4.46)	Destabilizing (+1.74)	Destabilizing (+1.62)
Congenital Hemolytic Anemia	S851F	-	-	Destabilizing (+21.16)	Destabilizing (+0.85)	Destabilizing (+0.83)
Congenital Hemolytic Anemia	P904L	-	-	Neutral (+0.14)	Neutral (+0.28)	Neutral (+0.47)
Congenital Hemolytic Anemia	I1024M	-	-	Neutral (-0.44)	Neutral (+0.39)	Neutral (+0.21)
Congenital Hemolytic Anemia	Q1062E	-	-	Destabilizing (+1.16)	Neutral (+0.18)	Neutral (+0.14)
	**ATP11A**					
Hearing Loss	S4N	-	-	Neutral (+0.22)	Neutral (-0.23)	Neutral (-0.07)
Leukodystrophy, hyopmylenation	A37V	-	-	Stabilizing (-0.58)	Neutral (-0.07)	Destabilizing (+0.64)
Leukodystrophy, hyopmylenation	Q84E	-	-	Neutral (-0.34)	Destabilizing (+1.91)	Neutral (+0.14)

*Reduced expression is defined as being ~<70% of WT protein expression levels

References

1.Takatsu, H. *et al.* Phospholipid Flippase Activities and Substrate Specificities of Human Type IV P-type ATPases Localized to the Plasma Membrane*. *Journal of Biological Chemistry* **289**, 33543–33556 (2014).

2.Folmer, D. E., van der Mark, V. A., Ho-Mok, K. S., Oude Elferink, R. P., and Paulusma, C. C. (2009) Differential effects of progressive familial intrahepatic cholestasis type 1 and benign recurrent intrahepatic cholestasis type 1 mutations on canalicular localization of ATP8B1. *Hepatology* 50, 1597-16052.

3.van der Velden, L. M., Stapelbroek, J. M., Krieger, E., van den Berghe, P. V., Berger, R., Verhulst, P. M., Holthuis, J. C., Houwen, R. H., Klomp, L. W., and van de Graaf, S. F. (2010) Folding defects in P-type ATP 8B1 associated with hereditary cholestasis are ameliorated by 4-phenylbutyrate. *Hepatology* **51**, 286-296

4. Liou, A. Y., Molday, L. L., Wang, J., Andersen, J. P. & Molday, R. S. Identification and functional analyses of disease-associated P4-ATPase phospholipid flippase variants in red blood cells. *J. Biol. Chem.* **294**, 6809–6821 (2019).

5. van Dijk, M. J., van Oirschot, B. A., Harrison, A. N., Recktenwald, S. M., Qiao, M., Stommen, A., Cloos, A. S., Vanderroost, J., Terrasi, R., Dey, K., Bos, J., Rab, M. A. E., Bogdanova, A., Minetti, G., Muccioli, G. G., Tyteca, D., Egee, S., Kaestner, L., Molday, R. S., van Beers, E. J., and van Wijk, R. (2023) A novel missense variant in ATP11C is associated with reduced red blood cell phosphatidylserine flippase activity and mild hereditary hemolytic anemia. *Am J Hematol (2023)* Sep 6. doi: 10.1002/ajh.27088. Epub ahead of print. PMID: 37671681.
